# Multimodal Transformer–Based Electrocardiogram Analysis for Cardiovascular Comorbidity Detection: Model Development and Validation Study

**DOI:** 10.2196/80815

**Published:** 2026-01-02

**Authors:** Zi Yang, Xiaojuan Wang, Jianlin Wang, Qi Guang, Xueqian Ding, Hao Liu, Yunpeng Xu, Jing Zhao, Ming Bai

**Affiliations:** 1 Information Center, The First Hospital of Lanzhou University, Lanzhou, China; 2 Heart Center, First Hospital of Lanzhou University, No. 1, Donggang West Rd., Chengguan Dist, Lanzhou, Gansu, 730000, China, 8609318356763; 3 The First School of Clinical Medicine of Lanzhou University, Lanzhou, China

**Keywords:** cardiovascular diseases, Electrocardiogram, multimodal deep learning, comorbidity detection, model interpretability, ECG

## Abstract

**Background:**

Cardiovascular diseases remain the leading global cause of mortality, yet traditional electrocardiogram (ECG) interpretation shows subjective variability and limited sensitivity to complex pathologies.

**Objective:**

This study aims to address these challenges by proposing the Cardiovascular Multimodal Prediction Network (CaMPNet), a transformer-based multimodal architecture that integrates raw 12-lead ECG waveforms, 9-structured machine-measured ECG features, and demographic data (age and sex) through cross-attention fusion.

**Methods:**

The model was trained on 384,877 records from the Medical Information Mart for Intensive Care IV - Electrocardiogram Matched Subset database and evaluated across 12 cardiovascular disease labels. To further assess temporal robustness, a temporal external validation was performed using the most recent 10% of the data, withheld chronologically from model development.

**Results:**

On the internal test set, the model achieved a mean area under the curve (AUC) of 0.845 (SD 0.04) and area under the precision-recall curve of 0.489, outperforming the residual networks-ECG baseline (AUC=0.848 but *F*_1_*-*score=0.152) and all single-modality variants. Subgroup analyses demonstrated consistent performance across demographics (male AUC= 0.846 vs female=0.843; youngest quartile 0.884 vs oldest 0.811). CaMPNet retained moderate discriminative ability in temporal external validation with a mean AUC of 0.715 (SD 0.03) and area under the precision-recall curve of 0.298, although performance declined due to temporal distribution shifts. Despite this, major disease categories, such as atrial fibrillation, heart failure, and normal rhythm, maintained high AUCs (>0.84). Attention-based visualization revealed clinically interpretable patterns (eg, ST-segment elevations in ST-segment elevation myocardial infarction), and ablation experiments verified the model’s tolerance to missing structured inputs.

**Conclusions:**

CaMPNet demonstrates robust and interpretable multimodal ECG-based diagnosis, offering a scalable framework for comorbidity screening and continual learning under real-world temporal dynamics.

## Introduction

Cardiovascular diseases (CVDs) [1] remain the leading cause of death worldwide, accounting for approximately 17.9 million deaths annually (32% of all global deaths) [2-4]. As a noninvasive, cost-effective, and widely available diagnostic tool, the electrocardiogram (ECG) [5-7] plays a vital role in screening and monitoring cardiac conditions, such as arrhythmias and myocardial ischemia [8]. However, traditional ECG interpretation often relies on expert-driven heuristics or rule-based algorithms, which can struggle with noisy signals or atypical patterns, limiting their use in early or complex disease detection [6,9-12].

Recent advances in deep learning (DL) [13] have enabled more effective ECG analysis by learning complex temporal and morphological features directly from raw signals [14-18]. Architectures such as convolutional neural networks [19], long short-term memory [2,3,9], and transformers [20] have demonstrated impressive performance in arrhythmia classification [2], heart failure (HF) prediction [11,21], and atrial fibrillation detection [4]. The availability of large public datasets (eg, Massachusetts Institute of Technology - Beth Israel Hospital, Medical Information Mart for Intensive Care - Electrocardiogram [22], and Physikalisch-Technische Bundesanstalt - XL) has further supported model development and validation [10,23]. Building on this progress, DL-based approaches have begun to address the limitations of conventional analysis [24-27], identifying precursors to atrial fibrillation [2,3] and predicting conditions such as ventricular dysfunction with high sensitivity [2,11]. Methodological advances have also explored fusing electronic health record data with ECG signals to emulate multimodal diagnostic workflows [6,28].

Despite this progress, 3 critical gaps persist in current ECG-based artificial intelligence systems. First, most prior studies focus on single-disease classification, failing to address the reality that cardiovascular comorbidities are highly prevalent, particularly among elderly and critically ill patients [11]. Second, the majority of models rely solely on raw ECG waveforms, overlooking the structured, machine-measured ECG features (eg, interval timings and electrical axes) that are routinely generated by hospital acquisition systems. These parameters contain clinically meaningful morphological descriptors but are often discarded in end-to-end DL pipelines. Third, model evaluation is frequently restricted to small or single-center datasets, raising concerns regarding robustness and generalizability across diverse populations.

To bridge these gaps, this study aims to answer a central question: Can a unified multimodal learning framework that intelligently fuses raw ECG waveforms with structured clinical features improve the detection of multiple cardiovascular comorbidities compared to single-modality approaches? Specifically, we sought to develop a model capable of leveraging the complementary information in machine-measured parameters while remaining robust to the missing or noisy data inherent in real-world clinical settings.

Recent advances in ECG-based DL have demonstrated the capability of convolutional architectures, particularly residual networks (ResNet), to extract robust diagnostic features directly from raw waveform data. For example, the ResNet-based model proposed by Kalmady et al [29] leveraged a large population-level cohort to predict multiple cardiovascular conditions and established deep residual learning as an effective backbone for ECG waveform analysis. This model serves as an important benchmark, as it represents a strong waveform-only approach that captures temporal morphology but does not incorporate structured ECG measurements or demographic information. Moreover, most existing models, including the ResNet baseline, adopt late or simple feature fusion strategies and therefore cannot explicitly model cross-modal interactions between waveform and structured inputs. In contrast, our proposed framework introduces an early cross-attention mechanism that allows structured ECG features to guide the selection of clinically relevant waveform segments prior to encoding. This design enables richer multimodal representation learning and provides a principled advantage over waveform-only models and traditional fusion strategies. To allow for a fair comparison, we include the ResNet-ECG model [29] as a key baseline and contextualize its differences from our method in both architecture and modality integration.

In this study, we developed Cardiovascular Multimodal Prediction Network (CaMPNet), a transformer-based multimodal framework developed using the large-scale Medical Information Mart for Intensive Care IV - Electrocardiogram Matched Subset (MIMIC-IV-ECG) database [22]. Unlike conventional fusion methods, CaMPNet introduces a query-conditioned early fusion mechanism to efficiently retrieve relevant waveform information using structured features. We rigorously validate the model’s performance across multilabel classification tasks, assess its robustness to missing inputs, and demonstrate its interpretability through attention maps that align with physiological patterns. This work represents a significant step toward scalable, clinically deployable artificial intelligence for comprehensive cardiovascular risk assessment.

## Methods

### Datasets and Cohort Selection

We used a total of 384,877 standard 12-lead ECG recordings from the publicly available MIMIC-IV-ECG database [22,30,31] hosted on PhysioNet, jointly curated by Massachusetts Institute of Technology-Beth Israel Deaconess Medical Center. To ensure consistency in model development and diagnostic label interpretation, we focused on the 13 most prevalent diagnostic categories. The 13 diagnostic categories considered in this study comprised one “Normal” rhythm class and 12 CVD classes, atrial fibrillation, supraventricular or ventricular tachycardia, cardiac arrest, atrioventricular block, unstable angina, ST-elevation or non–ST-elevation, myocardial infarction, pulmonary embolism, hypertrophic cardiomyopathy, aortic stenosis or insufficiency, mitral valve prolapse or stenosis, pulmonary hypertension, and heart failure. These disease labels were derived from the corresponding *International Statistical Classification of Diseases and Related Health Problems 10th Revision* codes associated with each ECG recording.

Table 1 summarizes the baseline demographic and clinical characteristics of the study population, including the distribution of age, gender, and the prevalence of each diagnostic category. It also presents the sample counts and proportions of the 13 diagnostic categories, across the entire dataset and its designated training, validation, and test splits, providing insights into (1) the representational balance across disease categories, (2) the presence of rare or underrepresented conditions, and (3) the need for targeted strategies, such as weighted loss functions or resampling techniques to address class imbalance during model training.

**Table 1.** Baseline characteristics.

Characteristics	Values (N=384,877)
Sex, n (%)	
Female	183,073 (47.6)
Male	201,804 (52.4)
Age (years), mean (SD)	64.8 (17)
Clinical labels, n (%)	
Normal	151,476 (39.4)
Atrial fibrillation	123,898 (32.2)
Heart failure	122,681 (31.9)
ST-Elevation or non-ST-elevation myocardial infarction	45,116 (11.7)
Pulmonary hypertension	29,018 (7.5)
Supraventricular or ventricular tachycardia	24,980 (6.5)
Hypertrophic cardiomyopathy	21,398 (5.6)
Aortic stenosis or insufficiency	19,682 (5.1)
Mitral valve prolapse or stenosis	18,716 (4.9)
Atrioventricular block	18,057 (4.7)
Unstable angina	13,067 (3.4)
Pulmonary embolism	8524 (2.2)
Cardiac arrest	7593 (2)

### CaMPNet Overview

**Figure 1.**
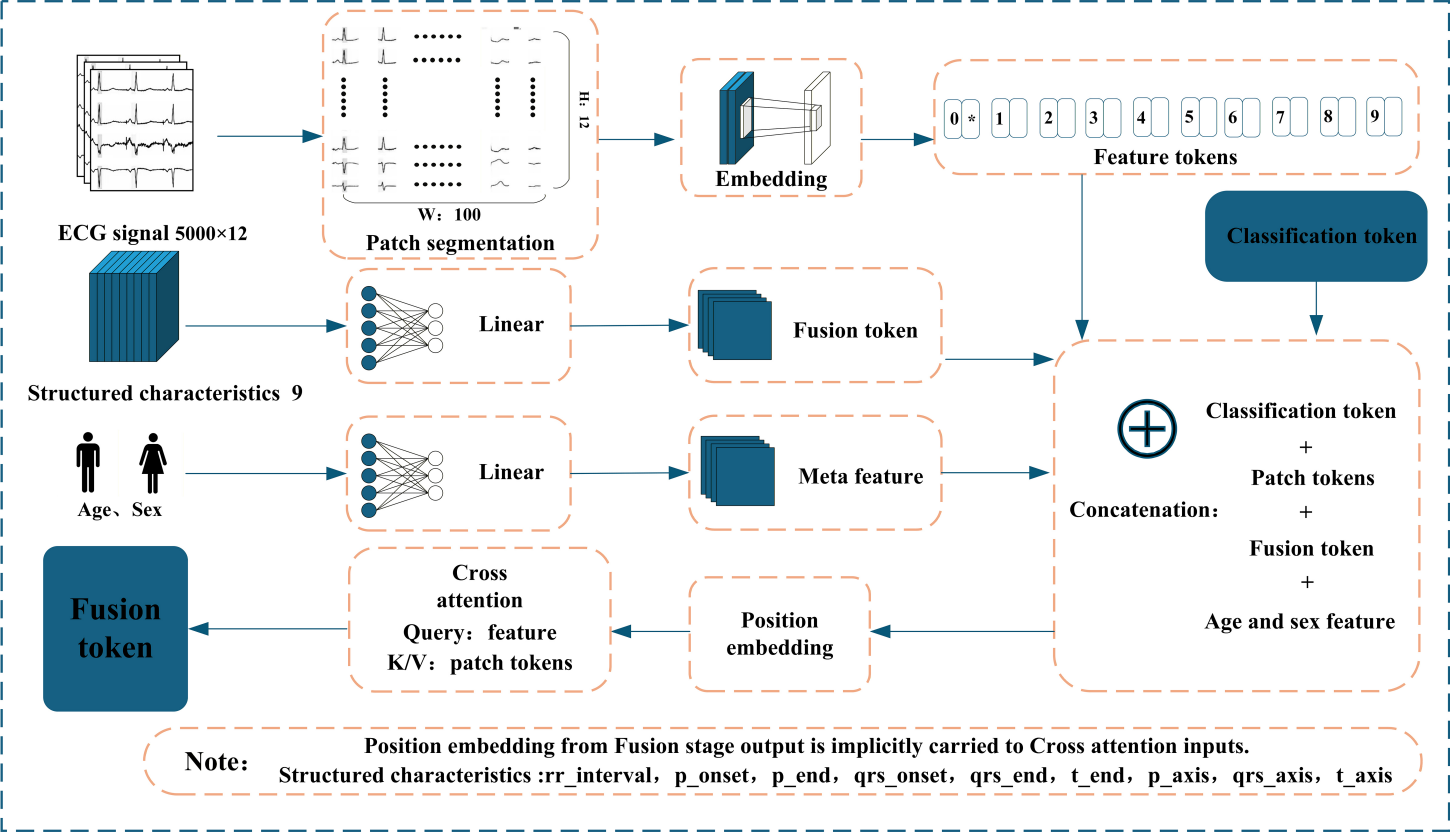
Overall architecture of the cardiovascular multimodal prediction network. The multimodal input embedding and fusion module jointly processes raw electrocardiogram waveforms, structured clinical features, and demographic metadata. A cross-attention mechanism generates a context-enhanced fusion token to integrate waveform and feature information. ECG: electrocardiogram; K/V: key value.

We propose CaMPNet, a novel multimodal transformer architecture designed for automatic classification of cardiac abnormalities. The model is specifically designed to embed and fuse heterogeneous data sources, including raw ECG waveforms, structured clinical features, and patient metadata, within a unified diagnostic framework. As shown in Figure 1, the model begins by processing the raw 12-lead ECG signals. They are divided into a series of nonoverlapping patches, which are then mapped into ECG patch tokens using a 1D convolutional network. This preserves essential temporal and morphological information from the waveforms. Meanwhile, structured clinical features and patient demographic information (age and sex) are each embedded into independent feature tokens via separate feed-forward networks, allowing the model to retain distinct semantics from each modality.

The core innovation of the CaMPNet lies in its early fusion strategy prior to the transformer encoder. Before entering the main transformer encoder, the model uses a cross-attention module, where the structured feature token acts as the query, actively retrieving and integrating the most relevant waveform information from the ECG patch token sequence (context), thereby generating an enhanced feature token enriched with low-level signal context.

**Figure 2.**
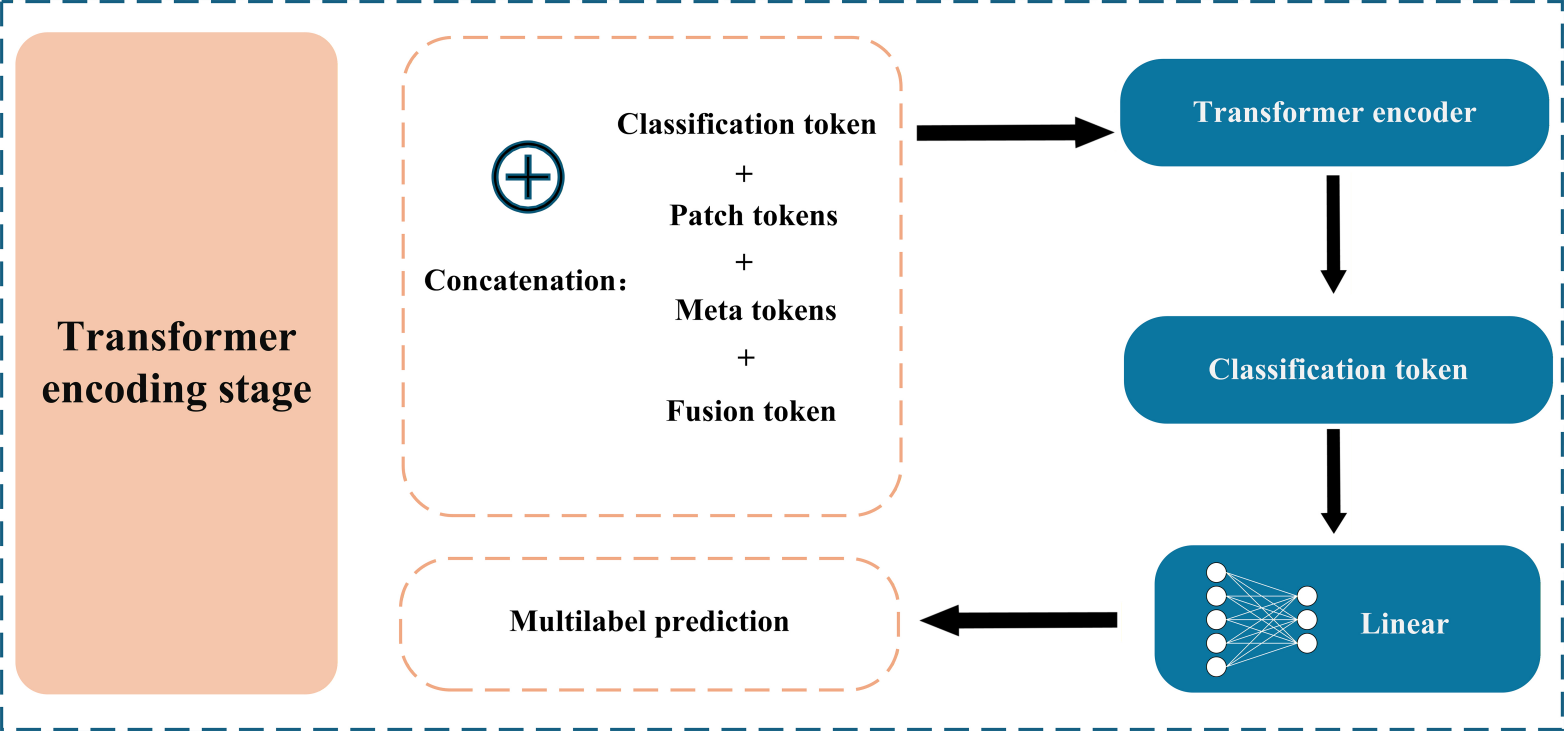
Transformer encoding and classification. The fused token sequence, including the electrocardiogram path tokens, fusion token, metadata token, and CLS token, is passed through a multilayer transformer encoder. The output from the CLS token is used for final multilabel cardiac classification.

Subsequently, the fusion token is concatenated with the original ECG patch tokens, metadata tokens, and a CLS token. Positional encodings are then added to form the final input sequence. As illustrated in Figure 2, the resulting sequence is fed into a standard multilayer transformer encoder, which deeply models all information through global self-attention mechanisms. Finally, the output state of the CLS token is used by a linear classification head to predict cardiac abnormality categories. This fusion-before-encoding strategy based on hybrid attention allows the model to more precisely exploit multimodal information, thereby improving diagnostic applications.

The novelty of CaMPNet lies in its hierarchical cross-modal attention fusion, which closely mirrors a clinician’s diagnostic reasoning process by jointly analyzing waveform dynamics, structured clinical metrics, and patient context, while maintaining computational efficiency.

#### Mathematical Formulation of Cross-Attention Fusion

Let $X_{p}\in R^{B\times N_{p}\times D}$denote the ECG patch embeddings (context) and $X_{s}\in R^{B\times 1\times D}$the structured ECG feature token (query). The cross-attention mechanism is defined as:


(1) $$Q=X_{s}W_{Q},\;K=X_{p}W_{K},V=X_{p}W_{v}$$

(2) $$A=Softmax\left(\frac{QK^{T}}{\sqrt{D}}\right),Z=AV$$

(3) $$\tilde{X}=ln\left(X_{s}+FFN\left(Z\right)\right)$$

where *W*_Q_, *W*_K_, and *W*_V_ are learnable projection matrices, and LN denotes layer normalization. In this design, the structured ECG feature token serves as the query, while ECG patch tokens provide key-value context. This allows the model to selectively retrieve waveform regions that are physiologically relevant to each structured feature (eg, QRS widening, focusing on QRS complexes), thereby achieving clinically interpretable multimodal fusion.

### Experiment and Design

This study aimed to develop and evaluate CaMPNet, a transformer-based multimodal DL framework capable of automatically distinguishing normal ECGs and 12 distinct cardiac abnormalities through the integration of raw 12-lead ECG waveforms, derived structured ECG features, and demographic metadata.

A comprehensive evaluation was performed in five methodological stages: (1) standardized data preparation and preprocessing; (2) model training with systematic hyperparameter optimization; (3) validation through cross-validation techniques; (4) final performance assessment on an independent test set to ensure unbiased diagnostic evaluation; and (5) temporal external validation, designed to assess the model’s robustness over time and simulate real-world deployment scenarios. Because acquiring an independent external validation cohort was highly challenging, existing ECG repositories either lacked confirmed diagnostic annotations or missed key structured clinical variables (9 tabular features in our framework). We followed a temporal split strategy similar to that used in the study by Gustafsson et al [26] on myocardial infarction prediction in emergency departments.

Specifically, the dataset was divided chronologically, and the earliest 90% of samples were used for model development (further split into training, validation, and internal testing), while the most recent 10% were reserved as a temporally separated test cohort. This design enabled evaluation of the model’s temporal generalization under potential distributional shifts, providing a practical alternative to external validation when access to fully labeled multimodal data was limited.

### Data Preprocessing

The dataset was first chronologically divided into 2 partitions to enable temporal generalization analysis: the earliest 90% of records were used for model development, while the most recent 10% were reserved as a temporally separated external validation cohort. Within the development portion, data were further randomly split in a 7:1:1 ratio into training, validation, and internal test subsets to ensure robust model tuning and unbiased evaluation, following recommended practices [12]. This temporal split strategy was adopted to simulate real-world deployment scenarios and mitigate potential time-related data leakage.

Table 2 summarizes the distribution of the 13 diagnostic categories, comprising one “Normal” rhythm class and 12 CVD classes, across all dataset partitions (total, training, validation, test, and temporal test). Each cell reports both the sample count and its percentage within each subset. The table highlights a notable class imbalance, with normal, atrial fibrillation, and HF being the most prevalent, whereas rare but clinically significant conditions, such as pulmonary embolism and cardiac arrest, remain underrepresented, underscoring the necessity of adaptive loss weighting and resampling strategies during model training.

**Table 2.** Distribution of diagnostic labels across all dataset splits.

Description	Total, n (%)	Training, n (%)	Validation, n (%)	Test, n (%)	Temporal test, n (%)
Atrial fibrillation	123,898 (32.2)	86,100 (32)	12,404 (32.2)	12,315 (32)	13,079 (34)
Aortic stenosis or insufficiency	19,682 (5.1)	139,42 (5.2)	2021 (5.3)	2026 (5.3)	1693 (4.4)
Atrioventricular block	18,057 (4.7)	12,856 (4.8)	1830 (4.8)	1755 (4.6)	1616 (4.2)
Cardiac arrest	7593 (2)	5385 (2)	763 (2)	745 (1.9)	700 (1.8)
Hypertrophic cardiomyopathy	21,398 (5.6)	15,011 (5.6)	2152 (5.6)	2098 (5.5)	2137 (5.6)
Heart failure	12,2681 (31.9)	84,812 (31.5)	11,995 (31.2)	12,146 (31.6)	13,728 (35.7)
Mitral valve prolapse or stenosis	18,716 (4.9)	12,990 (4.8)	1966 (5.1)	1851 (4.8)	1909 (5)
Normal	151,476 (39.4)	106,371 (39.5)	15,105 (39.2)	15,219 (39.5)	14,781 (38.4)
Pulmonary embolism	8524 (2.2)	6076 (2.3)	847 (2.2)	845 (2.2)	756 (2)
Pulmonary hypertension	29,018 (7.5)	20,046 (7.4)	2908 (7.6)	2886 (7.5)	3178 (8.3)
ST-elevation or non-ST-elevation myocardial infarction	45,116 (11.7)	32,017 (11.9)	4567 (11.9)	4579 (11.9)	3953 (10.3)
Supraventricular or ventricular tachycardia	24,980 (6.5)	17,594 (6.5)	2515 (6.5)	2466 (6.4)	2405 (6.2)
Unstable angina	13,067 (3.4)	9196 (3.4)	1346 (3.5)	1333 (3.5)	1192 (3.1)

#### ECG Signal Processing

Raw 12-lead ECG signals were preprocessed by replacing missing values with zeros, resampling each record to 5000 samples (10 s at 500 Hz) through truncation or zero-padding, and applying per-lead z-score normalization (μ=0, σ=1).

#### Structured Feature Processing

Nine clinically derived ECG features were standardized using statistics computed from the training set only to avoid data leakage. Missing feature entries were imputed with zeros and accompanied by binary validity masks to explicitly indicate feature availability. Age values were further normalized to mitigate scale imbalance across modalities.

#### Label Processing

Diagnostic codes were converted into multihot vectors to support multilabel classification. The fixed sequence length of 5000 samples (10s at 500 Hz) was chosen to conform with the MIMIC-IV-ECG standard, in which most 12-lead ECGs are recorded at 500 Hz over 10-second intervals. This uniform input length ensured consistent model representation while preventing information loss from shorter signals through zero-padding when necessary.

### Experiment Setup

All experiments were implemented using PyTorch (v2.6; Meta AI) as the primary DL platform with standard scientific-computing toolkits. Input ECG sequences consisted of 5000 samples per lead and were divided into 50-sample nonoverlapping patches (100 patches per record). Together with structured features, these patches were projected into a 768-dimensional embedding space, consistent with the dimensionality used in Vision Transformer and Bidirectional Encoder Representations from Transformers architectures, to capture comprehensive temporal and morphological information. The model architecture comprised a 6-layer Transformer encoder with 8 attention heads per layer, jointly processing waveform and structured inputs. Training batches were constructed with random shuffling to promote generalization, while validation and test phases used fixed ordering for reproducibility. This configuration achieved an appropriate balance between computational efficiency and temporal integrity of ECG sequences.

### Training Protocol

#### Optimization and Training Strategy

Model optimization was conducted using an adaptive gradient–based algorithm with a carefully tuned learning rate and regularization to ensure stable convergence. Training proceeded for up to 200 epochs, with gradient clipping applied to prevent numerical overflow. Throughout training, model performance was continuously monitored on the validation set.

To more comprehensively reflect generalization ability, a combined performance indicator integrating both discriminative power and classification accuracy was used to guide the learning-rate scheduler and early stopping mechanism. When the improvement of this integrated metric plateaued, the learning rate was automatically reduced with a decay schedule that incorporated relative thresholds and cooldown periods to enhance stability.

Early stopping was triggered when no meaningful improvement was observed for several consecutive epochs, and the checkpoint corresponding to the best validation performance was retained as the final model. All experiments were performed on high-performance computing hardware equipped with NVIDIA RTX 4080 graphics processing units.

#### Class-Imbalanced Loss Function

To address label imbalance across disease categories, we used an adaptive weighted binary cross-entropy loss that dynamically adjusts class-specific positive weights according to the prevalence of each cardiac condition.


(4) $$L=-\frac{1}{C}\sum_{c=1}^{c}\left[w_{c}y_{c}log\sigma \left({\hat{y}}_{c}\right)+\left(1-y_{c}\right)log\left(1-\sigma \left({\hat{y}}_{c}\right)\right)\right]$$

Where σ(⋅) is the sigmoid function, $y_{c}$ the ground-truth label, ${\hat{y}}_{c}$ the predicted logit, and $w_{lc}=\left(N-ln_{c}\right)/\left(n_{lc}+l\varepsilon \right)$ the adaptive positive weight computed from the training-set statistics. This formulation effectively enhances sensitivity to minority cardiovascular conditions while maintaining balanced optimization.

#### Evaluation Protocol

The best-performing model identified from the validation phase was subsequently evaluated on the independent test set under inference mode with gradient computation disabled. Model predictions were generated for all samples in the validation, internal test, and temporally external test cohorts. Evaluation was performed consistently across all cohorts to ensure reproducibility and comparability.

To assess generalization, the first 90% of ECG records were used for model development and the most recent 10% for temporal external validation. Within the development cohort, data were split into training, validation, and internal test sets in a 7:1:1 ratio. This temporal division simulated real-world deployment and mitigated potential time-related data leakage.

### Statistical Analysis

The statistical analyses were performed to quantitatively evaluate model performance and interpretability. All metrics, including area under the precision-recall curve (AUPRC), *F*_1_-score, recall, precision, accuracy, and specificity, were computed per disease label and averaged across all 13 categories using the macro-average strategy. A combined metric (0.5×AUC+0.5×accuracy) was used to guide learning rate scheduling and early stopping during training. To assess calibration reliability, predicted probabilities were further evaluated using Platt scaling, and calibration curves were generated to visualize agreement between predicted and observed outcomes. Subgroup analyses were conducted by gender and age quartiles to examine demographic robustness and potential bias. All analyses were implemented in Python 3.11 (Python Software Foundation).

### Ethical Considerations

The MIMIC-IV-ECG database used in this study was developed by the Massachusetts Institute of Technology and Beth Israel Deaconess Medical Center. The data collection and archiving procedures were approved by the Institutional Review Boards of Massachusetts Institute of Technology and Beth Israel Deaconess Medical Center (institutional review board protocol number 2001P001699). Because the database consists of retrospective data that were fully deidentified in accordance with the Health Insurance Portability and Accountability Act Safe Harbor provisions, the requirement for individual informed consent was waived by the institutional review boards. No identifiable private information was accessed, and all analyses adhered strictly to the data privacy and confidentiality policies outlined by PhysioNet.

The authors completed the Collaborative Institutional Training Initiative Program for human subjects research (certification number: 67231809) and signed the PhysioNet Credentialed Data Use Agreement prior to accessing the database. The study used only publicly available, anonymized data, and therefore, no additional institutional ethical approval or participant compensation was required.

## Results

### Overall and Condition-Specific Performance

Our CaMPNet demonstrated robust diagnostic capability across all evaluation metrics on the independent test set, as summarized in Table 3. Specifically, CaMPNet achieved a mean area under the receiver operating characteristic (ROC) curve (mean AUC=0.845, SD 0.04), comparable to the clinically validated ResNet-ECG baseline (AUC=0.848), while substantially outperforming it in average precision (AUPRC=0.489 vs 0.398,+22.8%) [29], reflecting enhanced reliability in handling imbalanced class distributions. In terms of *F*_1_-score, CaMPNet yielded 0.501 compared to 0.152 for ResNet-ECG, corresponding to a relative gain of +229%, indicating significantly better balance between precision and recall. CaMPNet maintained a favorable trade-off between sensitivity and specificity, with recall=0.493 and precision=0.515, alongside strong overall accuracy=0.923 and specificity=0.949. These results demonstrate CaMPNet’s ability to provide both accurate and clinically meaningful findings.

ECG-based predictions suggest its potential use as a decision-support tool in multilabel cardiovascular diagnostics.

At the condition-specific level, the model exhibited consistently high discrimination for prevalent diagnoses. As shown in Table 4, CaMPNet achieved near-perfect classification for normal ECGs (AUC=0.963, AUPRC=0.931, and *F*_1_-score=0.877) and excellent performance for atrial fibrillation and HF (AUCs=0.907 and 0.887, respectively). However, performance degraded for rarer conditions, such as pulmonary embolism (AUPRC=0.217 and *F*_1_-score=0.295) and aortic stenosis or insufficiency (AUPRC=0.390 and *F*_1_-score=0.427), which is likely attributed to their subtle ECG manifestations and low sample prevalence in the dataset. These findings highlight the model’s robust generalization in common cardiovascular conditions while indicating challenges in capturing rare but critical pathologies.

**Table 4.** Classification performance of the Cardiovascular Multimodal Prediction Network on the independent test set, with per-condition metrics reported across 13 diagnostic categories, including area under the curve, area under the precision-recall curve, *F*_1_-score, recall, precision, accuracy, and specificity.

Labels	AUC^a^	AUPRC^b^	*F*_1_-score	Recall	Precision	Accuracy	Specificity
Atrial fibrillation	0.907	0.836	0.761	0.776	0.746	0.844	0.876
Aortic stenosis or insufficiency	0.832	0.390	0.427	0.435	0.419	0.939	0.966
Atrioventricular block	0.888	0.456	0.485	0.477	0.494	0.954	0.977
Cardiac arrest	0.797	0.276	0.336	0.285	0.410	0.978	0.992
Hypertrophic cardiomyopathy	0.849	0.473	0.501	0.475	0.530	0.948	0.976
Heart failure	0.887	0.781	0.727	0.747	0.707	0.823	0.857
Mitral valve prolapse or stenosis	0.771	0.300	0.347	0.316	0.385	0.943	0.975
Normal	0.963	0.931	0.877	0.913	0.844	0.899	0.890
Pulmonary embolism	0.794	0.217	0.295	0.251	0.358	0.974	0.990
Pulmonary hypertension	0.796	0.389	0.410	0.371	0.458	0.920	0.964
ST-elevation or non-ST-elevation myocardial infarction	0.880	0.620	0.595	0.605	0.586	0.902	0.942
Supraventricular or ventricular tachycardia	0.805	0.6376	0.400	0.381	0.422	0.927	0.964
Unstable angina	0.820	0.306	0.352	0.375	0.331	0.952	0.973
Mean (SD)	0.845 (0.04)	0.489 (0.03)	0.501 (0.04)	0.493 (0.02)	0.515 (0.03)	0.923 (0.07)	0.949 (0.06)

a AUC: area under the curve.

b AUPRC: area under the precision-recall curve.

### ROC Curve Analysis

**Figure 3.**
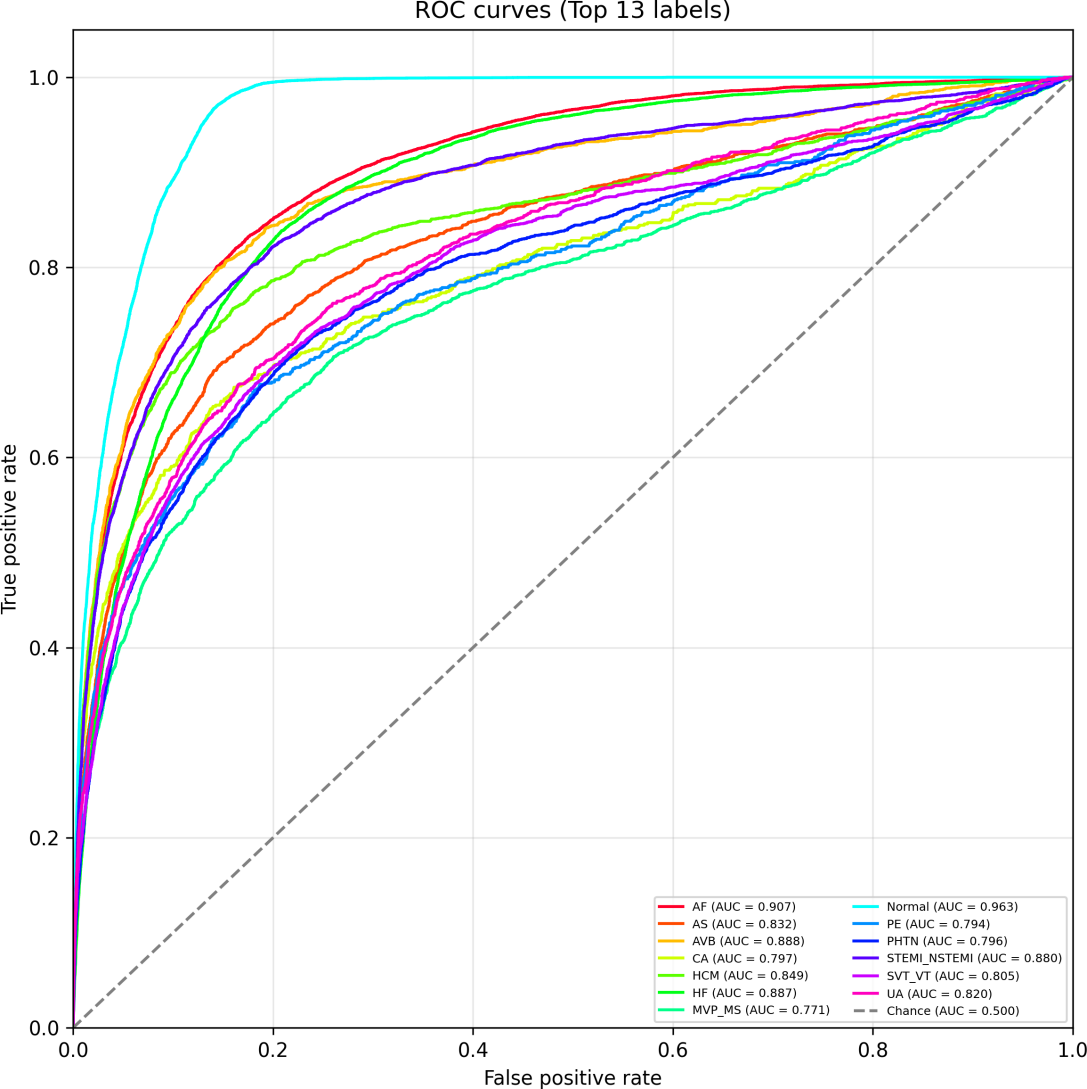
Receiver operating characteristic curves for the top 13 heart condition labels. Each curve shows the true positive rate (sensitivity) versus the false positive rate (1–specificity) across thresholds. The diagonal line denotes random chance (AUC=0.500). AF: atrial fibrillation; AS: aortic stenosis; AVB: atrioventricular block; CA: cardiac arrest; HCM: hypertrophic cardiomyopathy; HF: heart failure; MS: mitral stenosis; MVP: mitral valve prolapse; PE: pulmonary embolism; PHTN: pulmonary hypertension; STEMI-NSTEMI: ST-elevation myocardial infarction and non–ST-elevation myocardial infarction; SVT-VT: supraventricular tachycardia and ventricular tachycardia; UA: unstable angina.

To further illustrate the classification performance, we visualized the ROC curves of CaMPNet across the top 13 diagnostic categories, as shown in Figure 3. The ROC curve characterizes the relationship between the true positive rate (sensitivity) and the false positive rate (1–specificity) across varying decision thresholds, with the AUC serving as a comprehensive indicator of discriminative ability. The closer the AUC value is to 1.0, the better the model’s discriminative ability.

As illustrated in Figure 3, all plotted ROC curves substantially exceeded the diagonal line representing random chance (AUC=0.500), indicating that CaMPNet learned meaningful representations across all categories. Notably, the model achieved the highest AUC for the “Normal” class (AUC=0.963), reflecting its strong ability to distinguish normal cardiac activity. Other conditions with excellent AUCs included atrial fibrillation (AUC=0.907), atrioventricular block (AUC=0.888), and HF (AUC=0.887). These results confirm that the model is particularly effective in detecting common, well-represented cardiovascular conditions with distinct ECG signatures.

Meanwhile, for categories such as pulmonary embolism (AUC=0.794) and mitral valve prolapse or stenosis (AUC=0.771), the AUCs were slightly lower yet still demonstrated predictive value beyond random guessing. The shape and placement of the curves further reflect how performance varies at different operating points, offering clinically interpretable insights into the trade-offs between sensitivity and specificity per condition. Overall, the ROC analysis underscores CaMPNet’s strong and consistent ability to discriminate a broad range of cardiac conditions.

### Calibration Curve Analysis

**Figure 4.**
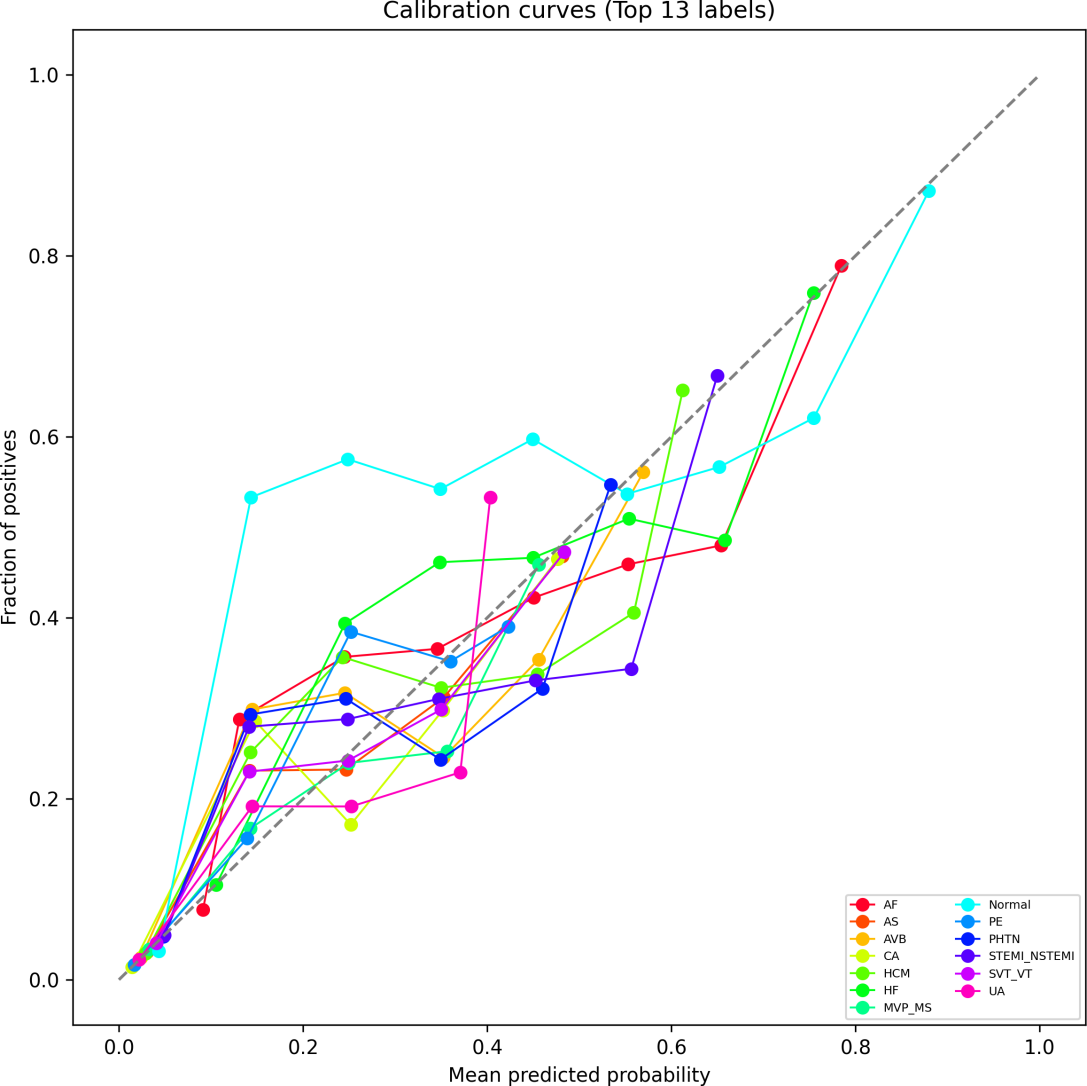
Calibration curves for the top 13 diagnostic categories. Each curve compares predicted probability versus the actual observed frequency. The diagonal line indicates perfect calibration. AF: atrial fibrillation; AS: aortic stenosis; AVB: atrioventricular block; CA: cardiac arrest; HCM: hypertrophic cardiomyopathy; HF: heart failure; MS: mitral stenosis; MVP: mitral valve prolapse; PE: pulmonary embolism; PHTN: pulmonary hypertension; STEMI-NSTEMI: ST-elevation myocardial infarction and non-ST-elevation myocardial infarction; SVT-VT: supraventricular tachycardia and ventricular tachycardia; UA: unstable angina.

While AUC quantifies discrimination, calibration curves provide insight into the reliability of predicted probabilities. As shown in Figure 4, we plotted calibration curves for the same 13 diagnostic categories to evaluate the agreement between predicted risk and actual observed frequency. Ideally, well-calibrated predictions should lie close to the diagonal line, where predicted probability equals empirical outcome frequency.

Despite some deviations, particularly in higher probability ranges, the overall upward trends of the curves confirm that the model’s output probabilities are not arbitrary but reflect meaningful likelihood estimates. This implies that CaMPNet can be trusted not only for classification decisions but also for risk stratification tasks where probability calibration matters.

### Subgroup Evaluation

To further assess the generalizability and fairness of CaMPNet across diverse patient populations, we conducted subgroup evaluations based on gender and age quartiles. Performance was measured using a comprehensive set of metrics, including AUC, AUPRC, *F*_1_-score, recall, precision, accuracy, and specificity.

### Gender Analysis

**Figure 5.**
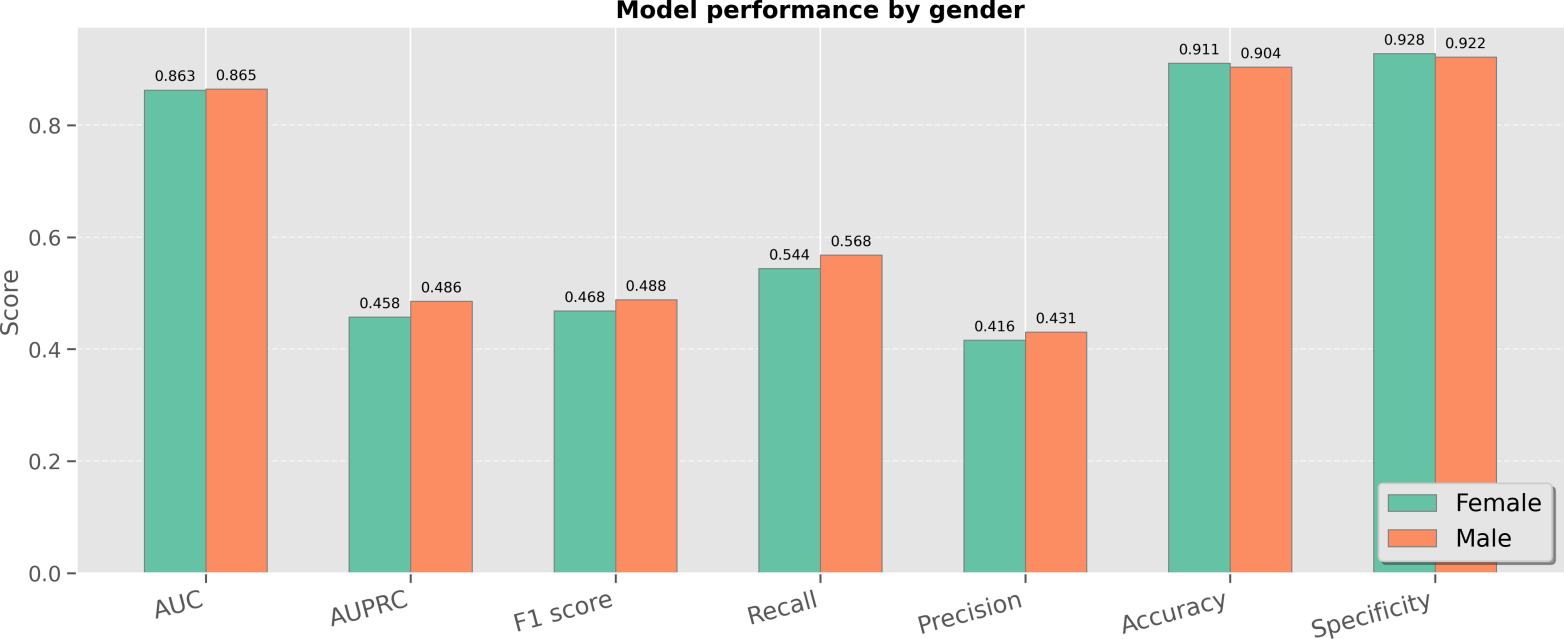
Model performance stratified by gender (male vs female), showing consistent diagnostic results and minimal sex-related variation. AUC: area under the curve; AUPRC: area under the precision-recall curve.

As illustrated in Figure 5, the model exhibited highly consistent performance across male and female subgroups. Specifically, AUC values were similar for females (0.838) and males (0.848); AUPRC was 0.465 for females and 0.502 for males, *F*_1_-score was 0.480 for females and 0.513 for males, recall values were 0.466 for females and 0.509 for males, precision scores were 0.500 (female) and 0.521 (male), accuracy remained high in both groups—0.927 for females and 0.920 for males, and specificity was similarly high at 0.952 (female) versus 0.946 (male). These results demonstrate that CaMPNet performs robustly and fairly across genders, showing minimal variability and no significant performance degradation, which suggests low susceptibility to sex-related bias in multilabel ECG-based prediction tasks.

### Age Stratification

**Figure 6.**
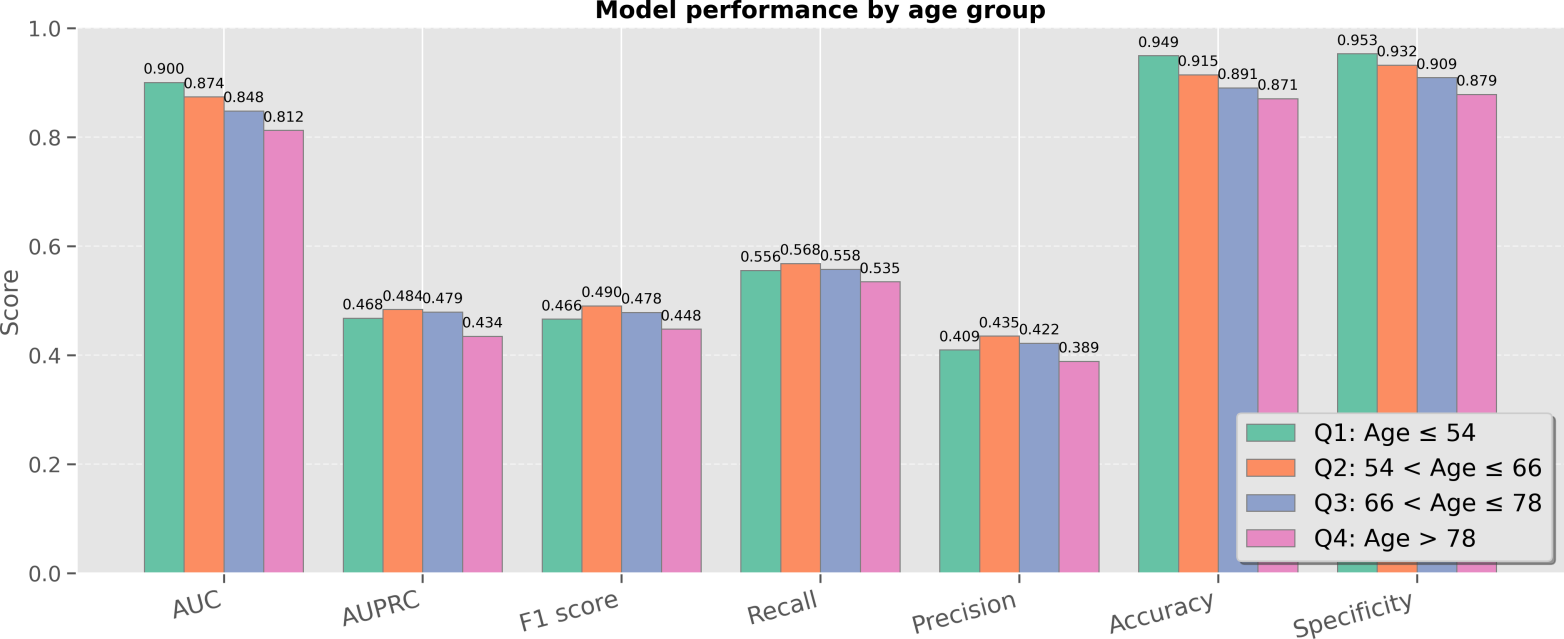
Model performance stratified by age quartiles (Q1: Age ≤54, Q2: 54‐66, Q3: 66‐78, Q4: Age >78). A slight performance decline was observed in the oldest group. AUC: area under the curve; AUPRC: area under the precision-recall curve.

We also stratified model performance by patient age using quartile splits, as shown in Figure 6.

The results suggest a subtle but notable trend: The Q1 group (age ≤54 y) achieved the highest performance across several metrics (AUC=0.872, AUPRC=0.496, *F*_1_-score=0.515, recall=0.524, precision=0.511, accuracy=0.956, specificity=0.964). The Q2 group (54<Age≤66 y) also performed well (AUC=0.847, AUPRC=0.486, *F*_1_-score=0.500, recall=0.496, precision=0.513, accuracy=0.928, specificity=0.952). The Q3 group (66 y<Age≤78 y) showed moderate performance (AUC=0.834, AUPRC=0.490, *F*_1_-score=0.502, recall=0.494, precision=0.515, accuracy=0.909, specificity=0.938). The Q4 group (Age>78 y) displayed a slight performance drop (AUC=0.811, AUPRC=0.448, *F*_1_-score=0.457, recall=0.451, precision=0.468, accuracy=0.894, specificity=0.920). The gradual decline in performance with increasing age, especially in Q4, could be attributed to the greater clinical complexity in older patients, including increased comorbidities and more variable ECG morphologies. Despite this, the model maintained acceptable performance even in the oldest group, indicating robust generalization across age cohorts.

The subgroup analysis indicates that CaMPNet maintains equitable performance across genders and demonstrates strong generalization across age groups. While younger subgroups benefit from slightly better performance, the model’s predictive capacity in older patients remains clinically viable.

These findings suggest that targeted model calibration or feature refinement for specific age ranges may offer opportunities for further performance enhancement.

### Attention Mechanism Interpretation

Building upon the quantitative evaluation of model performance, we further explore how CaMPNet makes multilabel diagnostic decisions by interpreting its internal attention patterns. Specifically, we visualize the cross-attention distribution between structured clinical features and ECG signal patches, aiming to understand which temporal segments of the ECG the model focuses on when predicting specific cardiac conditions.

These visualizations highlight the model’s cross-attention mechanism, where structured feature tokens serve as queries attending to ECG patch tokens. The highlighted vertical bands in each ECG plot indicate areas with relatively high attention weights—darker shades may correspond to stronger attention. Through case-based analysis, we reveal how attention is distributed across ECG cycles, providing insight into the model’s interpretability and potential clinical reasoning.

Figure S1 in Multimedia Appendix 1 presents a representative 12-lead ECG recording. The true and predicted labels are identical, “heart failure, ST-Elevation or Non-ST-Elevation myocardial infarction, supraventricular or ventricular tachycardia”, indicating a fully correct classification. Dark vertical bands denote ECG segments receiving stronger attention from the structured-feature token. The highlighted regions correspond primarily to the QRS complexes and adjacent ST segments across multiple precordial and limb leads (notably leads I, II, III, and V3-V5). These regions coincide with morphological features characteristic of acute ischemic injury and ventricular conduction abnormalities, manifested as widened QRS complexes, ST-segment elevations, and secondary repolarization changes. The network’s focus on these segments suggests that it captures both ischemic (ST-Elevation/Non-ST-Elevation Myocardial Infarction) and ventricular rhythm (supraventricular or ventricular tachycardia) signatures while also integrating the broader waveform flattening and amplitude modulation associated with HF-related remodeling.

Figure S2 in Multimedia Appendix 1 depicts another ECG recording with true labels “atrial fibrillation, hypertrophic cardiomyopathy” and model predictions “atrial fibrillation, hypertrophic cardiomyopathy.” Compared with Figure S1 in Multimedia Appendix 1, the attention map is more compact and periodic, concentrating around recurrent QRS complexes with moderate intensity in leads II, V1, and V3-V5. The focused attention reflects stable fusion between waveform and structured tokens when comorbid noise is minimal. The model achieves fully correct predictions, confirming that consistent rhythm morphology enables more decisive cross-attention alignment between structural ECG features and waveform segments.

Figure S3 in Multimedia Appendix 1 demonstrates an example where the true labels are “atrial fibrillation, cardiac arrest, hypertrophic cardiomyopathy, HF, ST-elevation or non-ST-elevation myocardial infarction” and the model predicts “Atrial fibrillation, aortic stenosis or insufficiency, cardiac arrest, hypertrophic cardiomyopathy, HF, ST-elevation or non-ST-elevation myocardial infarction.” Attention peaks align with the QRS and ST-segment elevations in precordial leads V1–V4, consistent with acute anterior ischemia. The model accurately detects ST-elevation or non-ST-elevation myocardial infarction and major comorbidities but adds mild over-prediction (aortic stenosis or insufficiency), reflecting partial confusion between ischemic and valvular patterns. The broader distribution of attention compared with Figures S1 and S2 Multimedia Appendix 1 indicates the model’s attempt to capture multiple concurrent abnormalities, revealing both its interpretability and current limitations in disentangling overlapping disease signatures.

In summary, these case studies demonstrate that attention visualization can effectively elucidate how CaMPNet integrates multimodal information to reach its diagnostic decisions. The highlighted regions reveal that the model consistently attends to physiologically meaningful waveform segments, particularly QRS complexes and ST-segment deviations, providing interpretable cues that align well with established clinical features. At the same time, the visualizations expose residual limitations: attention occasionally spreads across overlapping pathologies, leading to partial or excessive predictions in complex comorbidity cases. Together, these observations underscore both the interpretability and the current boundaries of CaMPNet’s reasoning process, motivating the discussion on its strengths and limitations in real-world multilabel ECG classification.

### Ablation Studies and Comparative Analysis

**Figure 7.**
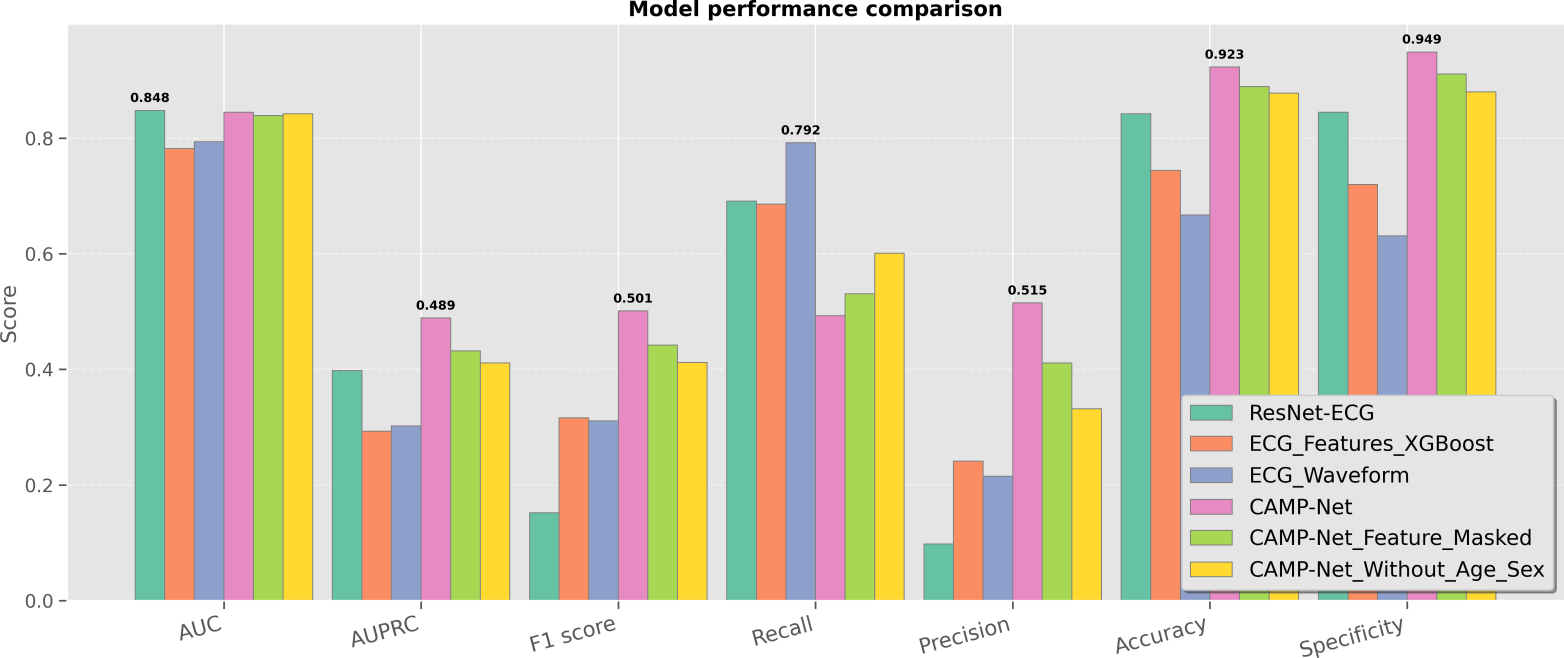
Model performance comparison across key metrics for cardiovascular multimodal prediction network, baseline models, and ablated variants. The cardiovascular multimodal prediction network achieves the highest area under the precision-recall curve, *F_1_*-score, precision, accuracy, and specificity, demonstrating its superior multimodal fusion capability. AUC: area under the curve; AUPRC: area under the precision-recall curve; CAMP-Net: consistency-aware multi-prior network; ECG: electrocardiogram; ResNet: residual neural network; XGBoost: extreme gradient boosting.

To systematically evaluate the contributions of each component within the proposed multimodal fusion model (CaMPNet) and benchmark its performance against related methods, we conducted a series of ablation experiments and comparative analyses. All models were trained and tested under identical data splits and evaluation metrics, with detailed configurations described previously. The results, summarized in Table 3, demonstrate the overall superiority of CaMPNet across multiple key metrics, compared to both unimodal and baseline models. A visual summary of the same results is provided in Figure 7, where a grouped bar chart clearly highlights CaMPNet’s superior performance across 7 evaluation metrics.

**Table 3.** Performance comparison of the Cardiovascular Multimodal Prediction Network and baseline models across multiple evaluation metrics.

Models	AUC^a^	AUPRC^b^	*F*_1_-score	Recall	Precision	Accuracy	Specificity
ResNet-ECG^cd^	0.848	0.398	0.152	0.691	0.098	0.842	0.845
ECG_Features_XGBoost^e^	0.782	0.293	0.316	0.686	0.241	0.744	0.72
ECG_Waveform	0.794	0.302	0.311	0.792	0.215	0.667	0.631
CaMPNet^f^^g^	0.845	0.489	0.501	0.493	0.515	0.923	0.949
CaMPNet_Feature_Masked	0.839	0.432	0.442	0.531	0.411	0.889	0.911
CaMPNet_Without_Age_Sex	0.842	0.411	0.412	0.601	0.332	0.878	0.88

a AUC: area under the receiver operating characteristic curve

b AUPRC: area under the precision-recall curve.

c ResNet: residual network.

d ECG: electrocardiogram.

e XGBoost: extreme gradient boosting.

f CaMPNet: Cardiovascular Multimodal Prediction Network.

g Proposed model used in this study.

In the following, we first present a detailed performance comparison across different model variants, highlighting the advantages of CaMPNet in terms of classification effectiveness.

### Overall Performance Comparison

CaMPNet achieved the highest overall performance across all evaluation metrics on the independent test set. Specifically, it attained an AUC of 0.845, comparable to the baseline ResNet-ECG (AUC=0.848), while substantially outperforming it in other metrics, such as AUPRC and *F*_1_-score. Compared with the waveform-only model ECG_Waveform (AUC=0.794) and the structured feature-based model ECG_Features_extreme gradient boosting (AUC=0.782), CaMPNet maintained superior balance across all evaluation criteria. In terms of average precision, CaMPNet achieved the best AUPRC of 0.489, indicating enhanced capability to handle class imbalance and improve confidence in positive sample recognition. It also reached the top *F*_1_-score of 0.501, reflecting a well-balanced trade-off between sensitivity (recall=0.493) and precision (precision=0.515). While the ECG_Waveform model exhibited the highest recall (0.792), CaMPNet demonstrated notably better precision, resulting in fewer false positives and more reliable positive predictions. Moreover, CaMPNet achieved the best accuracy (0.923) and specificity (0.949) among all models, confirming its robustness and superior diagnostic reliability across diverse evaluation criteria. These performance trends are clearly visible in Figure 7, where CaMPNet consistently appears as the leading bar in most metrics.

Taken together, these results highlight that CaMPNet achieves large relative improvements over the strong baseline ResNet-ECG. As shown in Table 3, while the AUC of CaMPNet (0.845) remained comparable to ResNet-ECG (0.848), it achieved a +229% increase in *F*_1_-score (0.501 vs 0.152), a +23% gain in AUPRC (0.489 vs 0.398), and a remarkable +425% boost in precision (0.515 vs 0.098). Overall accuracy and specificity also improved by +9.6% and +12.3%, respectively, reflecting enhanced reliability in distinguishing disease from normal conditions. Although recall decreased moderately (−29%), this trade-off was compensated by substantially higher precision, resulting in much stronger overall discriminative performance under class-imbalanced conditions. These relative improvements underscore the robustness and efficiency of the proposed multimodal cross-attention fusion mechanism compared to conventional convolutional neural network–based ECG classifiers.

### Robustness and Contribution of Structured Clinical Features

Considering the common issue of missing structured clinical features in real-world data, we further evaluated CaMPNet’s robustness by keeping the ECG waveform unchanged and zero-masking all 9 structured features at inference (denoted as CaMPNet_Feature_Masked in Table 3). The masked model exhibited only minor performance degradation, with an AUC decrease of 0.006 (from 0.845 to 0.839), AUPRC reduction of 0.057 (from 0.489 to 0.432), and *F*_1_-score drop of 0.059 (from 0.501 to 0.442), confirming the model’s tolerance to incomplete structured information. As shown in Figure 7, CaMPNet_Feature_Masked (green bars) remains close to the full CaMPNet variant across nearly all metrics, reinforcing the quantitative findings. Notably, even with masked features, CaMPNet_Feature_Masked still outperformed all unimodal models across most evaluation metrics, underscoring the crucial contribution of structured features during multimodal training. The cross-attention mechanism within the transformer encoder enables ECG feature extraction to leverage high-level semantic cues from structured variables, enhancing noise resilience and focusing on clinically salient pathological patterns. This multimodal interaction strengthens ECG representations, maintaining accurate and stable predictions even under feature absence at inference.

### Contribution of Demographic Attributes (Age and Sex)

To further investigate the role of demographic information, specifically age and sex, within the multimodal fusion framework, we conducted an ablation experiment in which both features were removed during training and inference (denoted as CaMPNet_Without_Age_Sex in Table 3).

The results show that the removal of demographic attributes led to slight performance degradation across all metrics, with AUC decreasing to 0.842, AUPRC to 0.411, and *F*_1_-score to 0.412. This marginal decline can also be observed in Figure 7, where the yellow bars representing CaMPNet_Without_Age_Sex remain close to those of the full CaMPNet, indicating only a subtle reduction across metrics. These findings suggest that age and sex provide useful prior knowledge for ECG-based diagnosis, yielding a modest yet consistent performance gain when included in the model. Notably, even without demographic inputs, CaMPNet_Without_Age_Sex still outperformed all baseline and unimodal models across most metrics, confirming that the model’s core advantage lies in the deep fusion between ECG waveform and structured clinical features, with demographic information serving as a valuable but auxiliary complement to this foundation.

In summary, these ablation and comparative analyses collectively demonstrate the strong complementarity between deep ECG representations and structured clinical variables. By systematically isolating each modality, we show that the cross-attention–based multimodal fusion module is the principal driver of CaMPNet’s performance gains and its robustness to incomplete data. Moreover, incorporating demographic attributes (age and sex) yields selective yet meaningful improvements across disease categories. The largest gains were observed in age- and sex-associated conditions, such as HF, hypertrophic cardiomyopathy, aortic stenosis or insufficiency, and pulmonary hypertension, while rhythm and ischemic disorders (eg, atrial fibrillation, supraventricular or ventricular tachycardia, ST-elevation or non–ST-elevation myocardial infarction) showed minimal changes. This indicates that demographic priors enrich the contextual understanding of chronic structural diseases rather than uniformly boosting all predictions.

Overall, the proposed architecture integrates richer and more nuanced discriminative cues, enabling superior and well-balanced results on the challenging multilabel cardiac abnormality task. These findings affirm the effectiveness of CaMPNet’s multimodal design and its key components in enhancing diagnostic accuracy, resilience, and overall clinical applicability.

### Temporal External Validation

To further assess the temporal robustness and real-world generalization of the proposed model, a temporal external validation was conducted using the most recent 10% of the available ECG data.

This subset was chronologically separated from the model development data to simulate prospective deployment conditions, ensuring that no temporal information leakage occurred between training and evaluation phases.

Compared with the internal test results, the overall performance of CaMPNet decreased, as shown in Table 5, when applied to the temporally separated cohort, indicating a noticeable temporal distribution shift between historical and recent ECG data. The mean AUC dropped from 0.845 (SD 0.04) to 0.715 (SD 0.03), and the mean AUPRC from 0.489 (SD 0.03) to 0.298 (SD 0.02), accompanied by reductions in *F*_1_-score (from 0.501 to 0.322) and accuracy (from 0.923 to 0.906). Despite this decline, diagnostic categories with abundant and stable ECG patterns, such as normal, atrial fibrillation, and HF, maintained relatively strong discriminative ability (AUCs=0.969, 0.862, and 0.845, respectively).

**Table 5.** Performance comparison of cardiovascular multimodal prediction network on temporal external validation cohort across multiple evaluation metrics.

Label	AUC^a^	AUPRC^b^	*F*_1_-score	Recall	Precision	Accuracy	Specificity
Atrial fibrillation	0.862	0.771	0.703	0.707	0.699	0.797	0.843
Aortic stenosis or insufficiency	0.670	0.104	0.150	0.162	0.141	0.920	0.954
Atrioventricular block	0.772	0.231	0.314	0.313	0.316	0.943	0.970
Cardiac arrest	0.569	0.032	0.048	0.039	0.063	0.972	0.989
Hypertrophic cardiomyopathy	0.682	0.178	0.231	0.199	0.276	0.927	0.969
Heart failure	0.845	0.729	0.683	0.667	0.700	0.779	0.842
Mitral valve prolapse or stenosis	0.578	0.084	0.115	0.096	0.143	0.926	0.970
Normal	0.969	0.936	0.898	0.958	0.845	0.916	0.891
Pulmonary Embolism	0.654	0.065	0.119	0.099	0.149	0.971	0.989
Pulmonary hypertension	0.641	0.176	0.212	0.174	0.270	0.893	0.958
ST-elevation or non–ST-elevation myocardial infarction	0.779	0.373	0.417	0.437	0.399	0.874	0.925
Supraventricular or ventricular tachycardia	0.625	0.122	0.160	0.140	0.186	0.908	0.959
Unstable angina	0.658	0.075	0.131	0.132	0.131	0.946	0.972
Mean (SD)	0.715 (0.03)	0.298 (0.02)	0.322 (0.03)	0.317 (0.03)	0.332 (0.04)	0.906 (0.05)	0.941 (0.06)

a AUC: area under the curve.

b AUPRC: area under the precision-recall curve.

Conversely, low-prevalence or morphologically heterogeneous conditions, such as cardiac arrest, mitral valve prolapse, mitral valve prolapse or stenosis, and pulmonary embolism, exhibited pronounced degradation (AUC <0.65), reflecting the sensitivity of rare-class predictions to evolving clinical data distributions.

These findings suggest that while CaMPNet retains substantial diagnostic capacity over time, its performance can be affected by long-term temporal drift in ECG acquisition, instrumentation, and labeling practices. The results underscore the importance of periodic model recalibration and continuous monitoring when deploying multimodal ECG classifiers in clinical practice to ensure sustained reliability in dynamically changing hospital environments.

### Interactive Clinical Prototype for ECG-Based Diagnosis

To facilitate the practical application and convenient evaluation of the proposed multimodal cardiac abnormality prediction model, we developed a user-friendly online prediction interface. This interface aims to provide a straightforward platform for users to obtain diagnostic predictions based on individual ECG signals and clinical information.

#### Input Module

Users are prompted to upload a standard 12-lead ECG signal in a CSV format, where each lead contains 5000 sampling points. Additionally, users are required to manually enter 2 basic demographic attributes (age and sex). The interface also offers optional input fields for submitting a predefined set of 9 structured ECG-derived features.

#### Prediction Mechanism and Model Robustness

Upon submission, the system invokes the pretrained CaMPNet model to perform multimodal inference. A key feature of this system is its adaptability; even when the 9 structured features are not provided, the model can still generate valid predictions based solely on the ECG waveform and demographic information. While full input coverage (including all structured features) leads to the highest prediction accuracy, experimental results have shown that the model maintains stable performance even in the presence of missing structured data, demonstrating strong robustness to incomplete inputs.

#### Result Presentation and Interpretability

Once the “Predict” button is clicked, the right-hand panel displays the model’s multilabel prediction outcomes. To enhance interpretability, the uploaded 12-lead ECG waveform is rendered, overlaid with an attention heatmap. This visualization highlights specific ECG segments that were most influenced by the structured features during the cross-attention phase, revealing which regions of the ECG the model deemed most relevant for decision-making.

#### Clinician Feedback Collection via Experience-Based Rating System

To gather real-world clinical feedback and continuously improve model performance, a rating mechanism has been embedded into the interface. After reviewing the predictions and attention visualizations, users are invited to rate the model’s prediction quality on a 1‐ to 5-star scale. Simultaneously, the interface records the user’s self-reported clinical experience level. All feedback entries, including scores and experience levels, are stored in the backend database in JSON format. This allows the research team to analyze how users of different expertise levels perceive the model’s reliability, offering valuable insights for iterative model refinement and clinical readiness assessment.

The development of the interactive prediction interface not only validates the feasibility of deploying the proposed model in real-world settings but also provides clinicians and researchers with an intuitive tool to explore model behavior and interpret its decision-making logic. Through integrated attention-based visualizations, users can gain deeper insights into how the model reaches its predictions, an essential step toward building trust, promoting clinical adoption, and guiding future iterations of model refinement. These features bridge the gap between algorithmic predictions and human interpretability, fostering a more transparent and collaborative human-AI interaction paradigm in cardiology.

## Discussion

### Principal Findings

In this study, we developed CaMPNet, a transformer-based multimodal ECG diagnostic framework that integrates raw 12-lead waveforms, structured machine-measured ECG features, and demographic attributes through an early cross-attention fusion mechanism. Our main findings demonstrate that CaMPNet substantially improves multilabel cardiovascular comorbidity detection compared with strong unimodal baselines, achieving a higher AUPRC (+23%), markedly improved *F*_1_-score (+229%), and better overall diagnostic balance than the ResNet-ECG benchmark. The model also showed robust performance across demographic subgroups and retained moderate discriminative ability under temporal distribution shift. The novelty of our approach lies in its query-conditioned early fusion design, which allows structured features to selectively retrieve physiologically relevant waveform segments, yielding richer representations and clinically interpretable attention patterns. These findings highlight CaMPNet’s potential impact as a scalable, interpretable tool for comprehensive ECG-based screening in real-world clinical workflows, especially in settings where multimodal inputs vary in completeness.

### Limitations

Despite the promising performance of the proposed CaMPNet in multimodal ECG-based multilabel classification tasks, several limitations remain. Unlike prior multimodal ECG systems that fuse by concatenation or shared self-attention, CaMPNet’s query-conditioned early cross-attention enforces directional information flow (structure → waveform) and natively tolerates missing inputs via validity-aware embeddings, which we found to be key to both robustness and interpretable retrieval. Although CaMPNet is designed for comorbidity detection, case analyses (Figure 7) show occasional omission or misclassification of concurrent conditions. This limitation arises from label co-occurrence sparsity and overlapping ECG manifestations. Future work will incorporate comorbidity-aware loss functions (eg, correlation-weighted binary cross-entropy) and graph-based label modeling to explicitly learn interdisease dependencies, enhancing the model’s clinical use in complex multimorbidity scenarios.

The model was trained and evaluated solely on 12-lead ECG samples from the MIMIC-IV-ECG database, which is predominantly sourced from intensive care unit settings in a North American tertiary medical center. To further assess robustness under temporal distribution shift, a temporal external validation was performed using the most recent 10% of data chronologically withheld from model development. Compared with the internal test set (mean AUC=0.845, SD 0.04; AUPRC=0.489), performance decreased in the temporal cohort (mean AUC=0.715, SD 0.03; mean AUPRC=0.298, SD 0.02; mean *F*_1_-score=0.322, SD 0.03). While CaMPNet maintained strong discriminative ability for common conditions, such as atrial fibrillation, HF, and normal rhythm, its accuracy declined for rare or morphologically variable disorders, suggesting sensitivity to evolving clinical practices and instrumentation. These findings highlight the importance of continuous model recalibration and multicenter external validation to ensure sustained clinical reliability.

The dataset also shows substantial class imbalance conditions, such as pulmonary embolism, cardiac arrest, and mitral valve disease, which were underrepresented, leading to relatively poor performance on these rare classes. Although class-weighted loss functions were applied, they cannot fully mitigate data scarcity in minority categories.

CaMPNet leverages 9 structured ECG features through a cross-attention mechanism to enhance predictive accuracy, yet these features may be missing or inconsistently measured in real-world clinical settings. Masking and zero-filling strategies provide partial robustness, but extensive or systematic missingness may still affect reliability. Moreover, the extraction of these features depends on specific analytic pipelines and acquisition devices, which may vary across hospitals and limit model portability.

While attention heatmaps improve interpretability by highlighting waveform segments attended by structured features, the internal reasoning of deep transformer models remains largely opaque. The current explanations do not always align with clinical diagnostic reasoning. Future work should incorporate expert domain knowledge and physiological priors into the interpretability module to improve transparency and clinical trust.

Finally, the dataset primarily represents adult patients in the intensive care unit with relatively homogeneous acquisition hardware and sampling rates. The model’s performance on pediatric populations, diverse geographic regions, and ECGs recorded at different sampling frequencies (eg, 250 Hz or 1000 Hz) remains untested. Future evaluations across multi-institutional, demographically diverse, and device-heterogeneous datasets will be essential to establish population-level generalizability.

### Future Directions

To support broader clinical adoption of CaMPNet, several directions merit future exploration. First, large-scale external validation across multiple centers and heterogeneous populations is needed to rigorously assess generalizability in real-world clinical environments. A major direction for future work is to further enhance model performance on rare but clinically critical cardiac conditions, such as pulmonary embolism, cardiac arrest, and mitral-valve prolapse, mitral valve prolapse or stenosis, which currently show limited recall and precision. Beyond the adaptive class-weighted loss used in this study, future efforts will explore more advanced imbalance-mitigation strategies, including focal or class-balanced focal loss to emphasize hard positive samples, synthetic data augmentation based on physiologically plausible waveform perturbations, and meta-learning or curriculum-based reweighting to dynamically adjust training focus. These approaches may help improve sensitivity to low-prevalence but high-impact pathologies and thus strengthen the model’s clinical applicability. Third, improving interpretability by aligning attention mechanisms with clinically recognized ECG features could enhance transparency and clinician trust. Finally, prospective clinical pilot studies incorporating user feedback will be essential to evaluate usability, guide interface design, and refine the model for deployment in routine health care workflows.

### Conclusions

This study demonstrates that CaMPNet provides a strong and clinically meaningful improvement in multimodal ECG-based diagnosis by effectively combining raw waveforms with structured ECG features through a novel cross-attention mechanism. The model achieved consistently higher precision, superior calibration behavior, and more balanced multilabel performance than conventional waveform-only architectures. Its robustness to missing structured features and stable performance across sex and age subgroups further support its use in real-world environments, where data completeness and patient heterogeneity are common challenges. The attention-based visualizations also offer an interpretable window into the model’s decision-making process, aligning learned representations with physiologic ECG patterns.

While several limitations remain, including rare-label performance, single-center data, and temporal distribution shifts, our findings underscore the strengths of the proposed multimodal framework and its potential as a foundation for clinically deployable ECG decision-support systems. Future efforts focusing on multicenter external validation, comorbidity-aware learning, and enhanced interpretability may further extend CaMPNet’s reliability and applicability in diverse health care settings.

## Supplementary material

10.2196/80815 Multimedia Appendix 1 Visualization of attention regions on three representative electrocardiograms alongside multi-label predictions and ground truth diagnoses.

## Abbreviations

AUC: area under the curve
AUPRC: area under the precision-recall curve
CaMPNet: cardiovascular multimodal prediction network
CVD: cardiovascular disease
DL: deep learning
ECG: electrocardiogram
HF: heart failure
HIPAA: Health Insurance Portability and Accountability Act
MIMIC-IV-ECG: Medical Information Mart for Intensive Care IV - Electrocardiogram Matched Subset
ResNet: residual network
ROC: receiver operating characteristic
