# Supplementary material for: Multimodal Transformer–Based Electrocardiogram Analysis for Cardiovascular Comorbidity Detection: Model Development and Validation Study
Source: JMIR Form Res. 2026 Jan 2;10:e80815. doi: 10.2196/80815 (PMC12758841; doi:10.2196/80815)

**Figure S1.** Attention visualization for a representative case with true label “heart failure, ST-Elevation or Non-ST-Elevation Myocardial Infarction, Supraventricular or Ventricular Tachycardia” and predicted “heart failure, ST-Elevation/Non-ST-Elevation Myocardial Infarction, Supraventricular or Ventricular Tachycardia.”


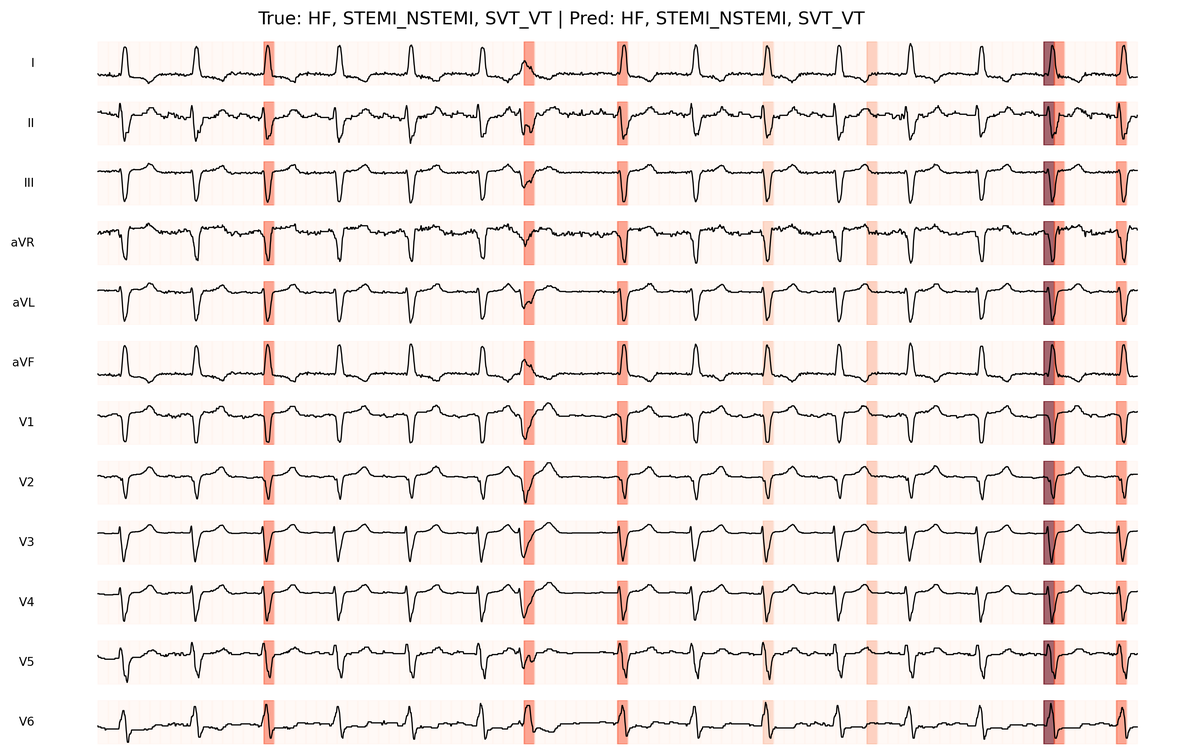


**Figure S2.** Attention map for a case with true label “Atrial Fibrillation, Hypertrophic Cardiomyopathy” and predicted “Atrial Fibrillation, Hypertrophic Cardiomyopathy.”


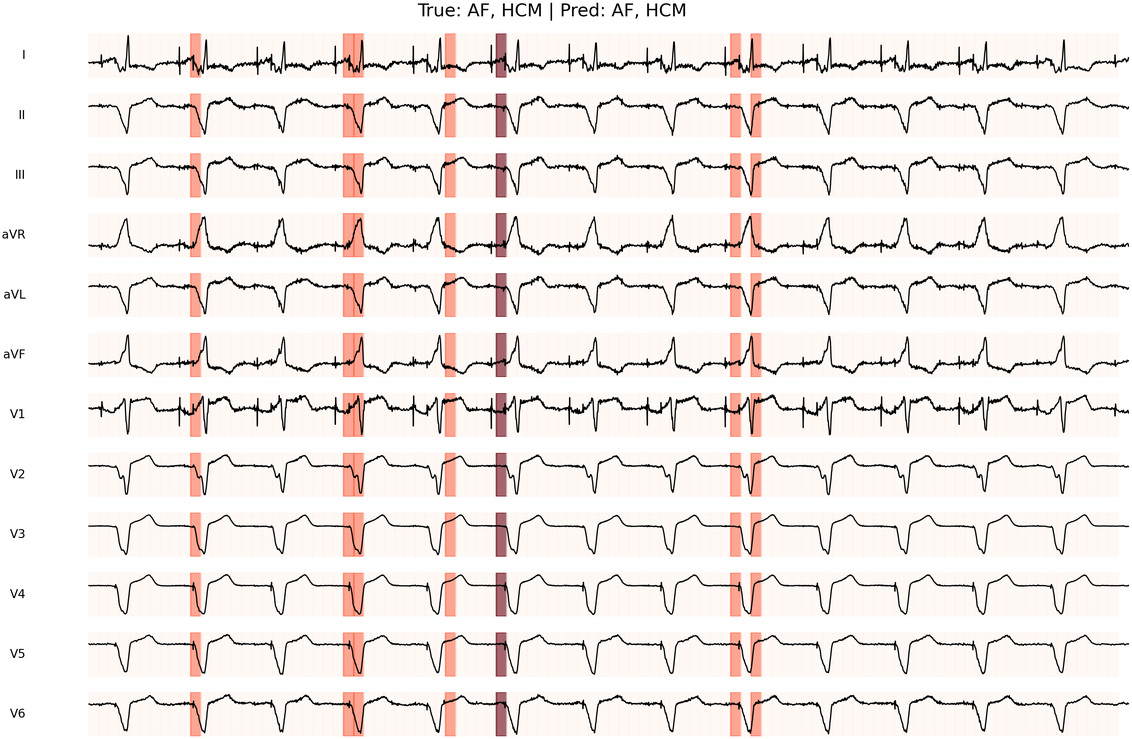


**Figure S3.** Attention map for a case with true label “Atrial Fibrillation, Cardiac Arrest, Hypertrophic Cardiomyopathy, heart failure, ST-Elevation or Non-ST-Elevation Myocardial Infarction” and predicted “Atrial Fibrillation, Aortic Stenosis/Insufficiency, Cardiac Arrest, Hypertrophic Cardiomyopathy, Heart Failure, ST-Elevation/Non-ST-Elevation Myocardial Infarction.”


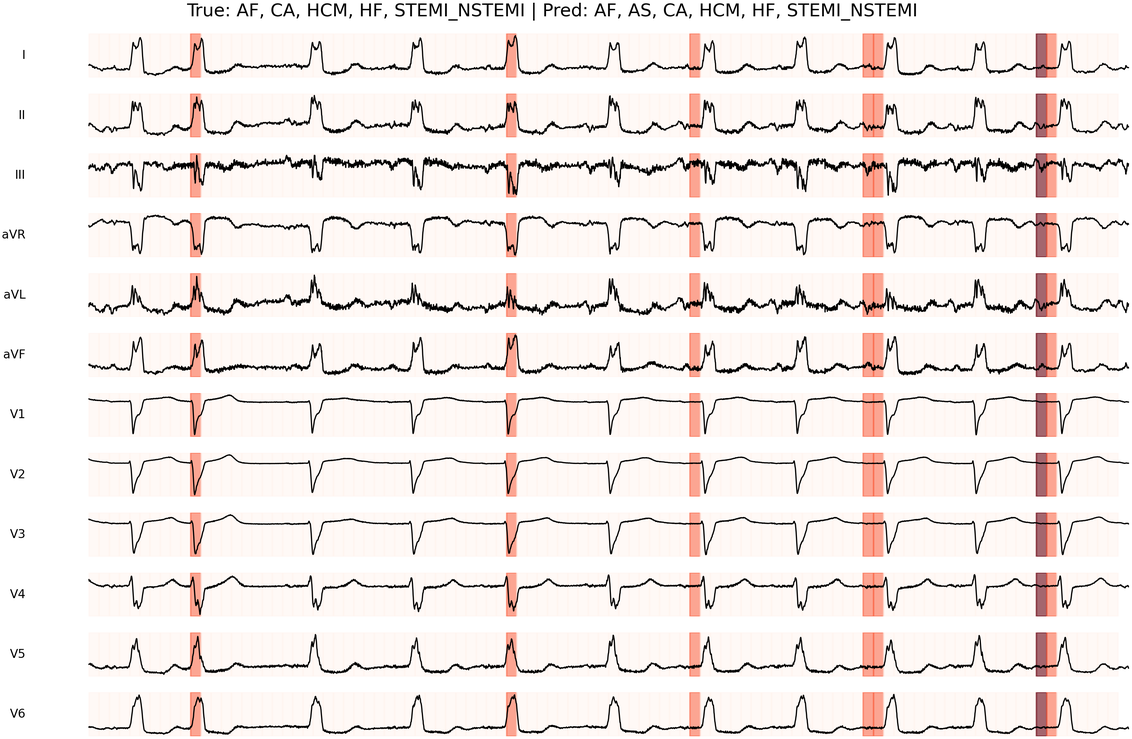

Supplement: Multimedia Appendix 1 [file formative-v10-e80815-s001.docx]
